# Cholesterol import and steroidogenesis are biosignatures for gastric cancer patient survival

**DOI:** 10.18632/oncotarget.13524

**Published:** 2016-11-23

**Authors:** Wei-Chun Chang, Shang-Fen Huang, Yang-Ming Lee, Hsueh-Chou Lai, Bi-Hua Cheng, Wei-Chung Cheng, Jason Yen-Ping Ho, Long-Bin Jeng, Wen-Lung Ma

**Affiliations:** ^1^ Sex Hormone Research Center, Department of Obstetrics and Gynecology, Department of Gastroenterology, Research Center for Tumor Medical Science, and Organ Transplantation Center, China Medical University/Hospital, Taichung, Taiwan; ^2^ Graduate Institute of Biomedical Sciences/Clinical Medical Science, School of Medicine, China Medical University, Taichung, Taiwan; ^3^ Department of Endocrinology and Metabolism, Changhua Christian Hospital, Changhua, Taiwan; ^4^ Department of OBs& GYN, Chia-Yi Chang-Gong Memorial Hospital, Chia-Yi, Taiwan; ^5^ Department of Nursing, Asia University, Taichung Taiwan

**Keywords:** cholesterol, steroidogenesis, gastric cancer, CYP19A1

## Abstract

Androgens, estrogens, progesterone and related signals are reported to be involved in the pathology of gastric cancer. However, varied conclusions exist based on serum hormone levels, receptor expressions, and *in vitro* or *in vivo* studies. This report used a web-based gene survival analyzer to evaluate biochemical processes, including cholesterol importing via lipoprotein/receptors (L/R route), steroidogenic enzymes, and steroid receptors, in gastric cancer patients prognosis. The sex hormone receptors (androgen receptor, progesterone receptor, and estrogen receptor ESR1 or ESR2), L/R route (low/high-density lipoprotein receptors, LDLR/LRP6/SR-B1 and lipoprotein lipase, LPL) and steroidogenic enzymes (CYP11A1, HSD3B1, CYP17, HSD17B1, HSD3B1, CYP19A1 and SRD5A1) were associated with 5-year survival of gastric cancer patients. The AR, PR, ESR1 and ESR2 are progression promoters, as are the L/R route LDLR, LRP6, SR-B1 and LPL. It was found that CYP11A1, HSD3B1, CYP17, HSD17B1 and CYP19A1 promote progression, but dihydrotestosterone (DHT) converting enzyme SRD5A1 suppresses progression. Analyzing steroidogenic lipidome with a hazard ratio score algorithm found that CYP19A1 is the progression confounder in surgery, HER2 positive or negative patients. Finally, in the other patient cohort from TCGA, CYP19A1 was expressed higher in the tumor compared to that in normal counterparts, and also promoted progression. Lastly, exemestrane (type II aromatase inhibitor) dramatically suppress GCa cell growth in pharmacological tolerable doses *in vitro*. This work depicts a route-specific outside-in delivery of cholesterol to promote disease progression, implicating a host-to-tumor macroenvironmental regulation. The result indicating lipoprotein-mediated cholesterol entry and steroidogenesis are GCa progression biosignatures. And the exemestrane clinical trial in GCa patients of unmet medical needs is suggested.

## INTRODUCTION

Gastric cancer (GCa) is the third leading cause of cancer death worldwide (World Health Organization, Cancer: Fact Sheet No 297; http://www.who.int/mediacentre/factsheets/fs297/en/). The high mortality rates of GCa are due to late diagnosis [1] and a lack of effective adjuvant therapy agents [2]. The prognosis and survival is very poor for advanced stage cancer patients receiving gastrectomy, with only a 30% 5-year survival rate [3]. However, there is also limited effective chemotherapy for early stage cancer patients [4, 5]. One meta-analysis review of surgery and chemotherapy in GCa patients reported a limited efficacy of current standard therapy [6]. Therefore, it is of great importance to find a novel strategy for GCa therapy.

Those who have GCa are predominantly male, which leads to speculation on the role sex hormones play in GCa development [7], although this is controversial within literatures. First, a large-scale epidemiological survey indicating female factors, e.g., reproductive age, ovariectomy surgery, breast-feeding, pregnancy, contraceptive agents, etc. suggests that the estrogenic signal suppresses the incidence of GCa [8–10]. On the other hand, there are several pieces of data that go against this opinion. For example, a large-scale survey (1299 patients) indicates that female factors contribute to poor survival of GCa, and male factors contribute to GCa patient survival following surgery [11]. Additionally, Progesterone Receptor (PR) expression is significantly upregulated in GCa tissue [12], although serum progesterone levels do not correlated with incidences of GCa [13]. Moreover, serum testosterone levels are significantly decreased in incidences of recurring GCa [14], and low testosterone levels are correlated with post-surgery complications [15]. The conflicting results between serum levels of sex hormones and receptor expression levels implicate a possible role of intrinsic de novo synthesis of sex steroids in GCa.

The initial biochemical process of steroidogenesis begins by converting cholesterol to pregnolone. Extracellular inflow is considered to be the major cellular cholesterol resource [16]. Lipoproteins, lipid carriers that engulf through the lipoprotein receptor, are one major route to provide cholesterol into cells. There are evidences suggesting that lipoprotein circulation might be the resource providing cholesterol to promote GCa. First, the cholesterol-rich lipid droplet is commonly observed in GCa lesions. Second, lipoprotein receptor is expressed in GCa or parental mucosa [17]. Third, the lipoprotein loading content could also affect GCa disease development [18]. Among lipoproteins, low-density lipoproteins (LDL) and high-density lipoprotiens (HDL) are major cholesterol carriers in circulation. Reports have documented a potential hazard of HDL and LDL in GCa incidences and progression [19]. Therefore, it would be suspicious if LDL and HDL were the cholesterol providers promoting GCa. This study hypothesized that circulating lipoproteins could carry cholesterol into cells to provide cellular needs; the gate for lipoprotein entrance (lipoprotein receptors) and the enzyme to release lipids from lipoproteins (Lipoprotein Lipase; LPL) could be a cancer facilitator as well [18]. The components involved in lipoprotein engulfing and cholesterol release is defined as Lipoprotein/Receptor route (L/R route) in this study. And the aim was to test the connectivity of L/R route to steroidogenesis, and, consequently, an altered GCa progression via action through related nuclear receptors.

To analyze the L/R route to steroidogenesis pathway in patients, web-based gene survival analysis was implemented in this study. The strategy uses meta-analysis of online cDNA microarray databases that predict the outcome in appropriately powered cohorts and provides a feasible, unbiased and genome-wide approach to analyze genes in cancer progression [20, 21]. There are online databases to validate the importance of gene expressions in GCa patient survival (http://kmplot.com/analysis/index.php?p=service&cancer=gastric). Here, we utilized a web-based survival analyzer (Kaplan-Meier plotter) to test candidate genes in GCa disease survival and calculate the importance of gene clusters in GCa patients of unmet medical needs. At last, we introduce CYP19A1 inhibitor in GCa cells to verify targeting effects.

## RESULTS

### Linking lipoprotein/receptor route to steroidogenesis in GCa progression

**Figure 1:**
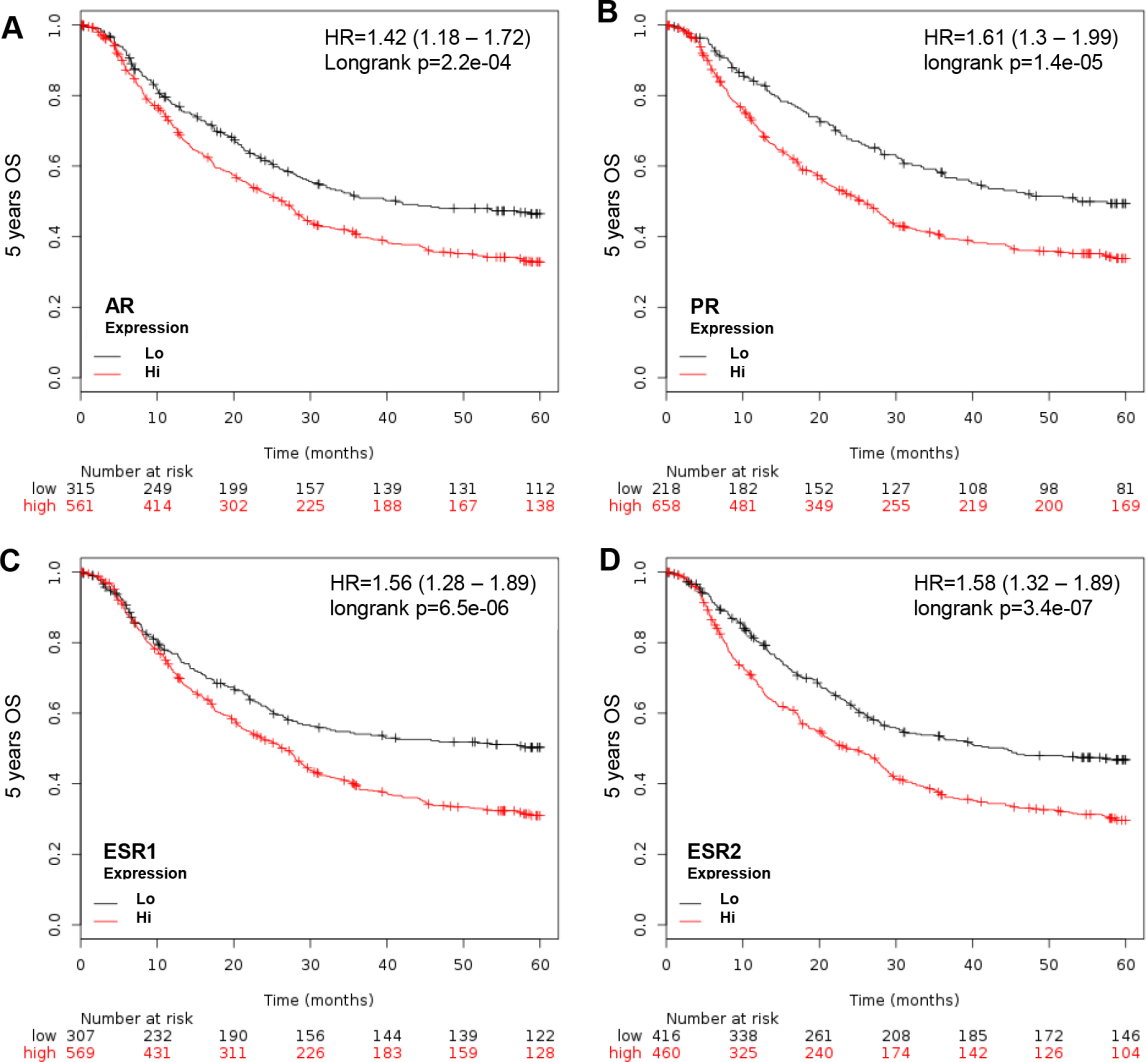
KM plotter evaluation of sex hormone nuclear receptors, including AR, PR, ESR1 and ESR2, in GCa 5-year OS (**A**) AR expression status in GCa patients. Red line indicates high expression and black line indicates low expression. At the initial time point (0 months), 561 patients have high AR and 315 have low AR. At the last time point (60 months), 138 have high AR and 112 have low AR. The HR is 1.42 (range 1.18–1.72), and *p*-value is 2.2e-04. (**B**) PR expression status in GCa patients. The number of 0 month patients with high PR is 658, and low PR is 218. The 60-month patient number with high PR is 169, and low PR is 81. The HR is 1.61 (range 1.3–1.99), and *p*-value is 1.4e-05. (**C**) ESR1 expression status in GCa patients. The 0 month patient number with high ESR1 is 569, and low ESR1 is 307. The 60-month patient number of high ESR1 is 128, and low ESR1 is 122. The HR is 1.56 (range 1.28–1.89), and *p*-value is 6.5e-06. (**D**) ESR2 expression status in GCa patients. The 0 month patient number of high ESR2 is 460, and low ESR2 is 307. The 60–month patient number of high ESR2 is 104, and low ESR2 is 146. The HR is 1.58 (range 1.32–1.89), and *p*-value is 3.4e-07.

The Kaplan Meier Survival Analyzer provides a platform to evaluate gene expression in GCa progression. The importance of sex steroid hormone nuclear receptor expression in 5-year OS of all GCa patients was weighted without differentiating between sexes. Four major nuclear receptors, including AR (androgen receptor), PR (progesterone receptor), ESR1 (estrogen receptor 1; ERα) and ESR2 (estrogen receptor 2; ERβ) are GCa progression promoter (Figure 1). The Hazard Ratios (HR) of each are: 1.42 (1.18-1.72; *p* = 2.2e-04; Figure 1A) for AR, 1.61 (1.3–1.99; *p* = 1.4e-05; Figure 1B) for PR, 1.56 (1 .28–1.89; *p* = 6.5e-06; Figure 1C) for ESR1 and 1.58 (1.32–1.89; *p* = 3.4e-07; Figure 1D) for ESR2. Therefore, the four nuclear receptors are independent prognosis markers in GCa, regardless of gender or serum hormone levels.

**Figure 2:**
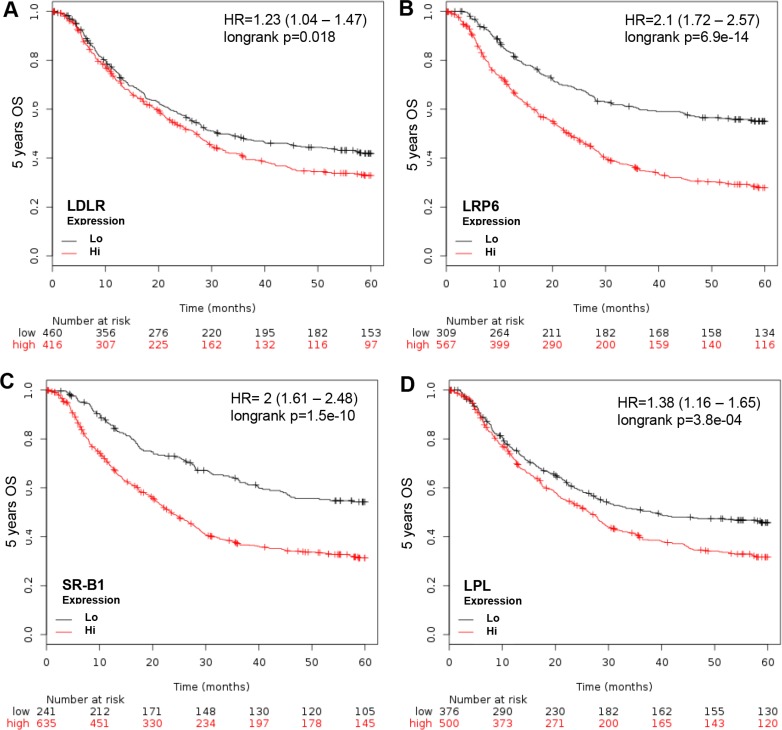
KM plotter evaluation of L/R route, including LDLR, LRP6, SR-B1 and LPL in GCa 5-year OS (**A**) LDLR expression status in GCa patients. Red line indicates high expression and the black line indicates low expression. The 0 month patient number with high LDLR is 416, and low LDLR is 460. The last 60-month patient number with high LDLR is 97, and low LDLR is 153. The HR is 1.23 (range 1.04–1.47), and *p*-value is 0.018. (**B**) LRP6 expression status in GCa patients. The 0 month patient number with high LRP6 is 567, and low LRP6 is 309. The 60-month patient number with high LRP6 is 116, and low LRP6 is 134. The HR is 2.1 (range 1.72–2.57), and *p*-value is 6.9e-14. (**C**) SR-B1 expression status in GCa patients. The 0 month patient number with high SR-B1 is 639, and low SR-B1 is 241. The 60-month patient number with high SR-B1 is 145, and low SR-B1 is 105. The HR is 2 (range 1.61–2.48), and *p*-value is 1.5e-10. (**D**) LPL expression status in GCa patients. The 0 month patient number with high LPL is 500, and low LPL is 376. The 60-month patient number with high LPL is 120, and low LPL is 130. The HR is 1.38 (range 1.16–1.65), and *p*-value is 3.8e-04.

To determine if the L/R route participates in GCa 5-year OS, LDLR, LRP6 (LDLR related protein 6) (26), SR-B1 (Scavenger receptor-B1, HDL receptor [22]) and LPL (lipoprotein lipase) were weighted in relation to 5-year OS. The HRs of each were: 1.23 (1.04-1.47; *p* = 0.018) for LDLR, 2.1 (1.72-2.57; *p* = 6.9e-14) for LRP6, 2 (1.61–2.48; *p* = 1.5e-10), and 1.38 (1.16–1.65; *p* = 3.8e-04) for LPL (Figure 2). This indicates that the L/R route shuttles cholesterol into GCa cells to facilitate cancer progression.

**Figure 3:**
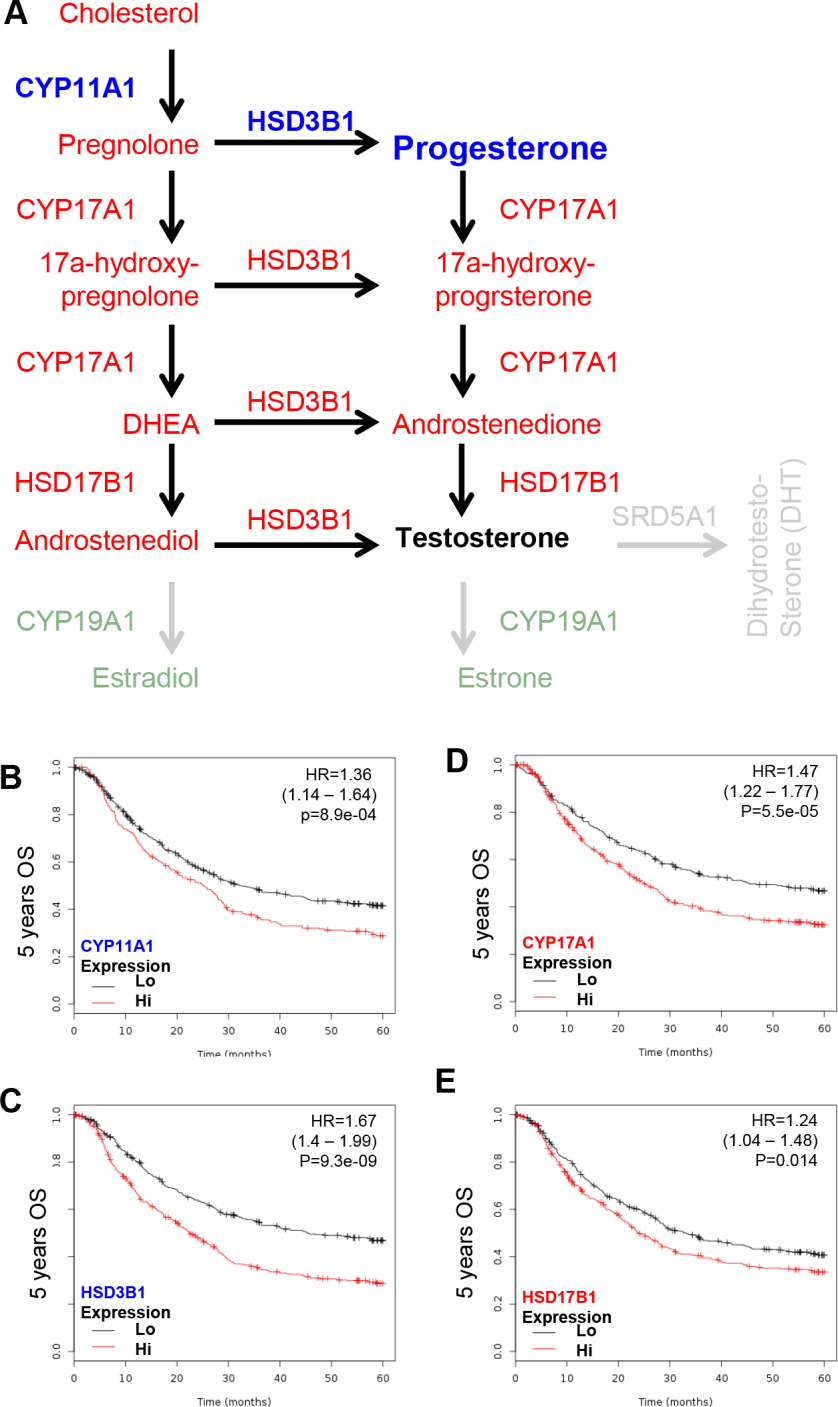
KM plotter evaluation of sex steroid lipidome related enzyme, including CYP11A1, CYP17, HSD3B1 and HSD17B1 in GCa 5-year OS (**A**) Schematic illustration of sex steroid lipidomes and responding genes, including CYP11A1 (conversion of cholesterol to pregnolone; blue colored), CYP17 (conversion between pregnolone, 17a-hydroxyprognolone, androstenedione, progesterone, 17a-hydroxy- progesterone and DHEA; red colored), HSD3B1 (conversion of pregnolone, 17a-hydroxyprognolone, or androstenedione to progesterone, 17a-hydroxyprogesterone, or DHEA; red colored) and HSD17B1 (conversion of DHEA or androstenedione to androstenediol or testosterone; red colored). (**B**) CYP11A1 expression status in GCa patients. The HR is 1.36 (range 1.14–1.64), and *p*-value is 8.9e-04. (**C**) HSD3B1 expression status in GCa patients. The HR is 1.67 (range 1.4–1.99), and *p*-value is 9.3e-09. (**D**) CYP17 expression status in GCa patients. The HR is 1.47 (range 1.22–1.77), and *p*-value is 5.5e-05. (**E**) HSD17B1 expression status in GCa patients. The HR is 1.24 (range 1.04–1.48), and *p*-value is 0.014.

Since the PR is a GCa progression independent promoter and the L/R route elevates cellular cholesterol content to promote GCa, the steroidogenic enzyme toward progesterone in GCa 5-years OS was examined. The CYP11A1 (cholesterol to pregnolone), CYP17 (pregnolone to 17α-hydroxy- pregnolone and Dihydroxyepiandrostendiol (DHEA)), HSD3B1 (pregnolone to progesterone; 17α-hydroxy-pregnolone to 17α-hydroxy-progesterone; DHEA to androstenedione; and androstenediol to testosterone) and HSD17B1 (DHEA to androstenediol) are involved in the production of progesterone (Figure 3A). Therefore, these four enzymes were adjusted and all were found to be GCa progression promoter. The HRs were 1.36 (1.14–1.64; *p* = 8.9e-04; Figure 3B) for CYP11A1, 1.47 (1.22–1.77; *p* = 5.5e-05; Figure 3D) for CYP17, 1.67 (1.4–1.99; *p* = 9.3e-09; Figure 3C) for HSD3B1 and 1.24 (1.04–1.48; *p* = 0.014; Figure 3E) for HSD17B1. These data indicate that progesterone production enzymes are GCa progression promoters. The pathological conversions of sex hormones from pregnolone to androstenediol and to testosterone are GCa progression favorable biochemical process.

**Figure 4:**
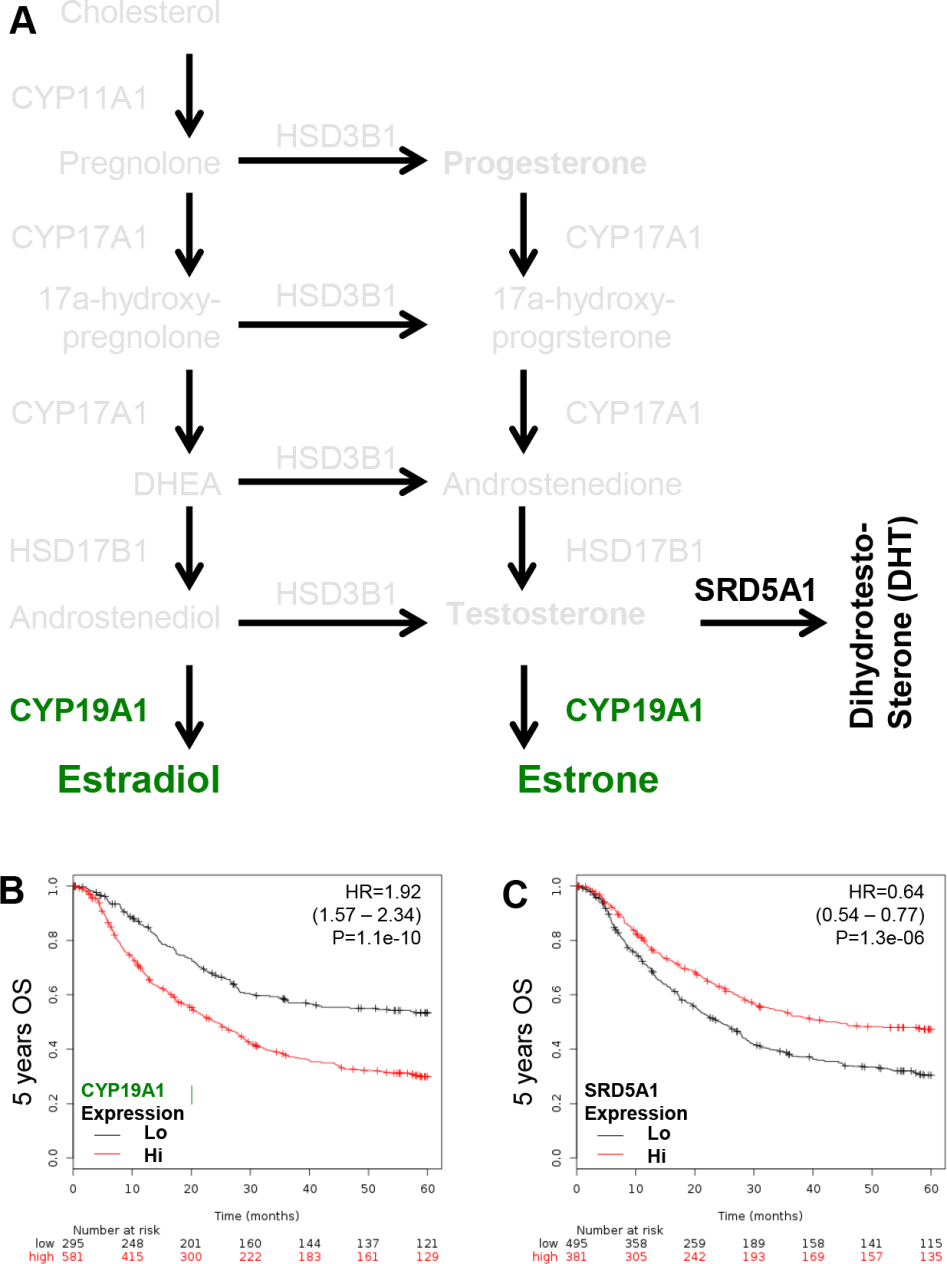
KM plotter evaluation of rate limiting step enzymes for estradiol (CYP19A1; aromatase) and DHT (SRD5A1; 5α-reductase) in GCa 5-years OS (**A**) Schematic illustration of estradiol and DHT lipidomes and responding genes, including CYP19A1 (conversion of androstenedione or testosterone to estradiol or estrone; green colored), SRD5A1 (conversion of testosterone to DHT; black colored). (**B**) CYP19A1 expression status in GCa patients. The HR is 1.92 (range 1.57–2.34), and *p*-value is 1.1e-10. (**C**) SR-B1 expression status in GCa patients. The HR is 0.64 (range 0.54–0.77), and *p*-value is 1.3e-06.

The critical enzymes governing the conversion of androstenediol or testosterone to estradiol (active form of estrogen; CYP19A1) and testosterone to dihydrotestosterone (DHT, active form of androgen; SRD5A1) were examined (Figure 4A). This was done to associate the ligand and receptor function in GCa. CYP19A1 is a poor GCa prognosis marker with HR = 1.92 (1.57 – 2.34; *p* = 1.1e-10; Figure 4B); however, SDR5A1 is good GCa prognosis marker with HR = 0.64 (0.54 – 0.77; *p* = 1.3e-06; Figure 4C). Thus, the pathological conversion of steroidogenesis in GCa is to favor progesterone and estradiol production, but not DHT.

Together, these data shows that the L/R route shuttles cholesterol into tumors, which is linked to steroidogenesis enzymes and to producing ligands for PR and ESRs to promote GCa progression. Further, DHT anabolism is not a favorable pathological biochemical process to promote GCa progression.

### Algorithm of HR score determined CYP19A1 could be novel target for GCa therapy

To score gene clusters responsible for de novo synthesis of progesterone, estradiol, or DHT, we utilized an algorithm (Formula 1) to test the importance of steroidogenic lipidomes in GCa. Current therapeutic regimens for GCa include surgery or chemotherapy [23]. Incomplete gastrectomy patients usually receive combined 5-fluouricil (5-FU) treatments as adjuvant chemotherapies [23, 24]. The median survival rate for patients undergoing surgery and 5-FU ranges from 36 to 91 months [24]. Anti-HER2 therapy has been introduced to HER2 positive (HER2+) GCa patients [25]. However, the anti-HER2 regimen exhibits only marginal survival benefits [25]. Therefore, understanding unmet medical needs of GCa will require evaluating patient subgroups that underwent surgery, surgery and 5-FU therapy and HER2 expression status. The KM plotter provides survival information for those subcategories of GCa patients.

**Figure 5:**
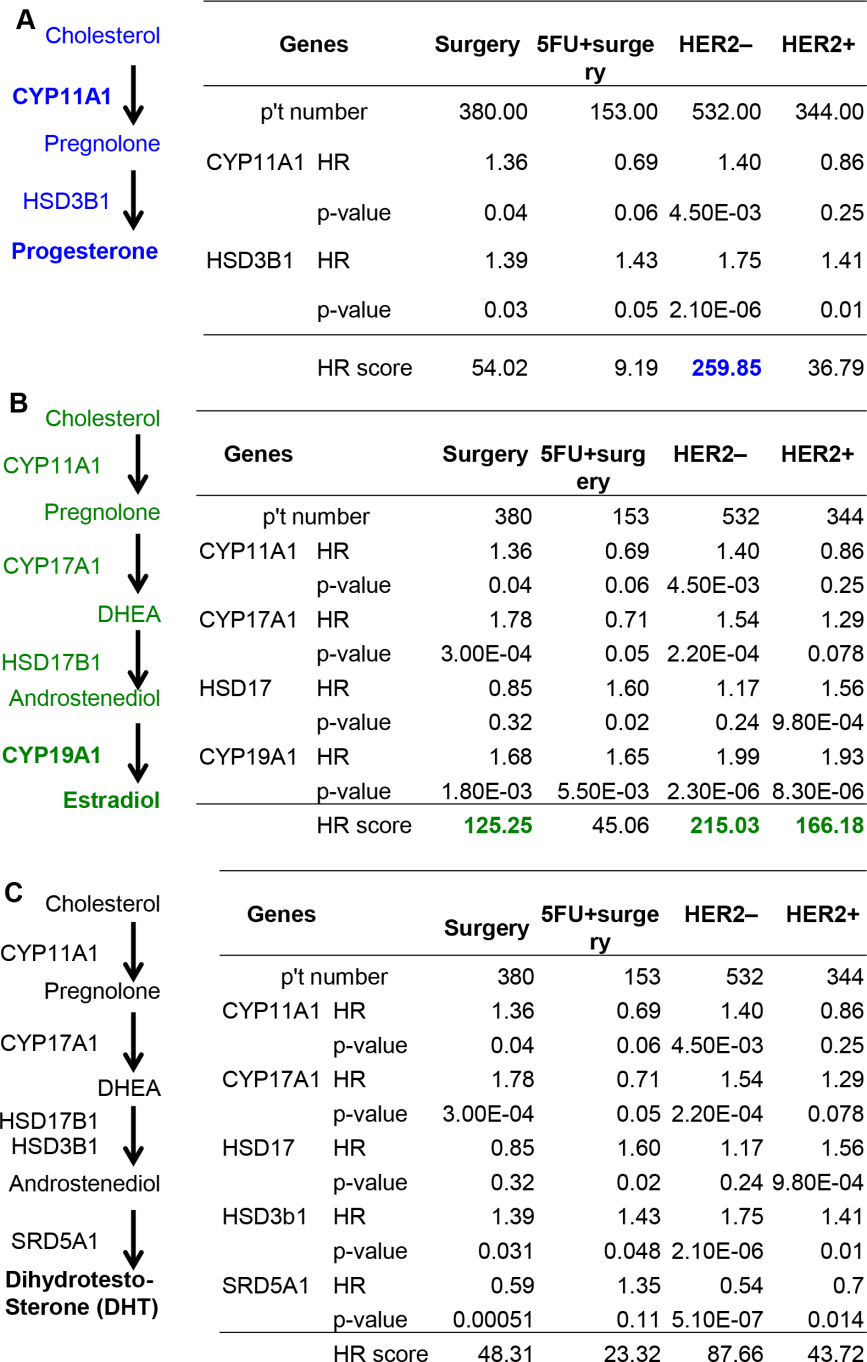
Calculation of the HR score of steroidogenic lipidomes in GCa progression (**A**) Progesterone anabolic enzymes CYP11A1 and HSD3B1 in surgery, 5-FU and surgery, HER2– or HER2+ subcategories of GCa patients. The HR score of each subcategory are: 54.02 for surgery, 9.19 for 5-FU and surgery, 259.85 for HER2– and 36.79 for HER2+ patients. (**B**) Estradiol anabolic enzymes CYP11A1, CYP17, HSD17B1 and CYP19A1 in the four subcategories of GCa patients. The HR score of each subcategory are: 125.25 for surgery, 45.06 for 5-FU and surgery, 215.03 for HER2– and 166.18 for HER2+ patients. (**C**) DHT anabolic enzymes CYP11A1, CYP17, HSD3B1 and SRD5A1 in four the subcategories of GCa patients. The HR score of each subcategory are: 48.31 for surgery, 23.32 for 5-FU and surgery, 87.66 for HER2– and 43.72 for HER2+ patients.

CYP11A1 and HSD3A1 are responsible for the production of progesterone, as shown in the anabolic pathway for progesterone (left panel, Figure 5A). The HR score for progesterone production is 54.02 in surgery, 9.19 in surgery and 5-FU, 259.85 in HER2 negative (HER2–), and 36.79 in HER2+ patients. The HR score in HER2– patients is high, which indicates targeting progesterone production might be effective in HER2– GCa patients. As shown in the anabolic pathway of estradiol production (left panel, Figure 5B), CYP11A1, CYP17, HSD17B1 and CYP19A1 are responsible for the production of estradiol. The HR score for estradiol production is 125.25 in surgery, 45.06 in surgery and 5-FU, 215.03 in HER2–, and 166.18 in HER2+ patients. Since the HR score in surgery, HER2+ and HER2– patients are high, targeting estradiol production might be effective. The anabolic pathway of DHT production shows that CYP11A1, CYP17, HSD17B1 and SRD5A1 are responsible for the production of DHT (left panel, Figure 3C). The HR score for DHT production is 48.31 in surgery, 23.32 in surgery and 5-FU, 87.66 in HER2–, and 43.72 in HER2+ patients.

**Figure 6:**
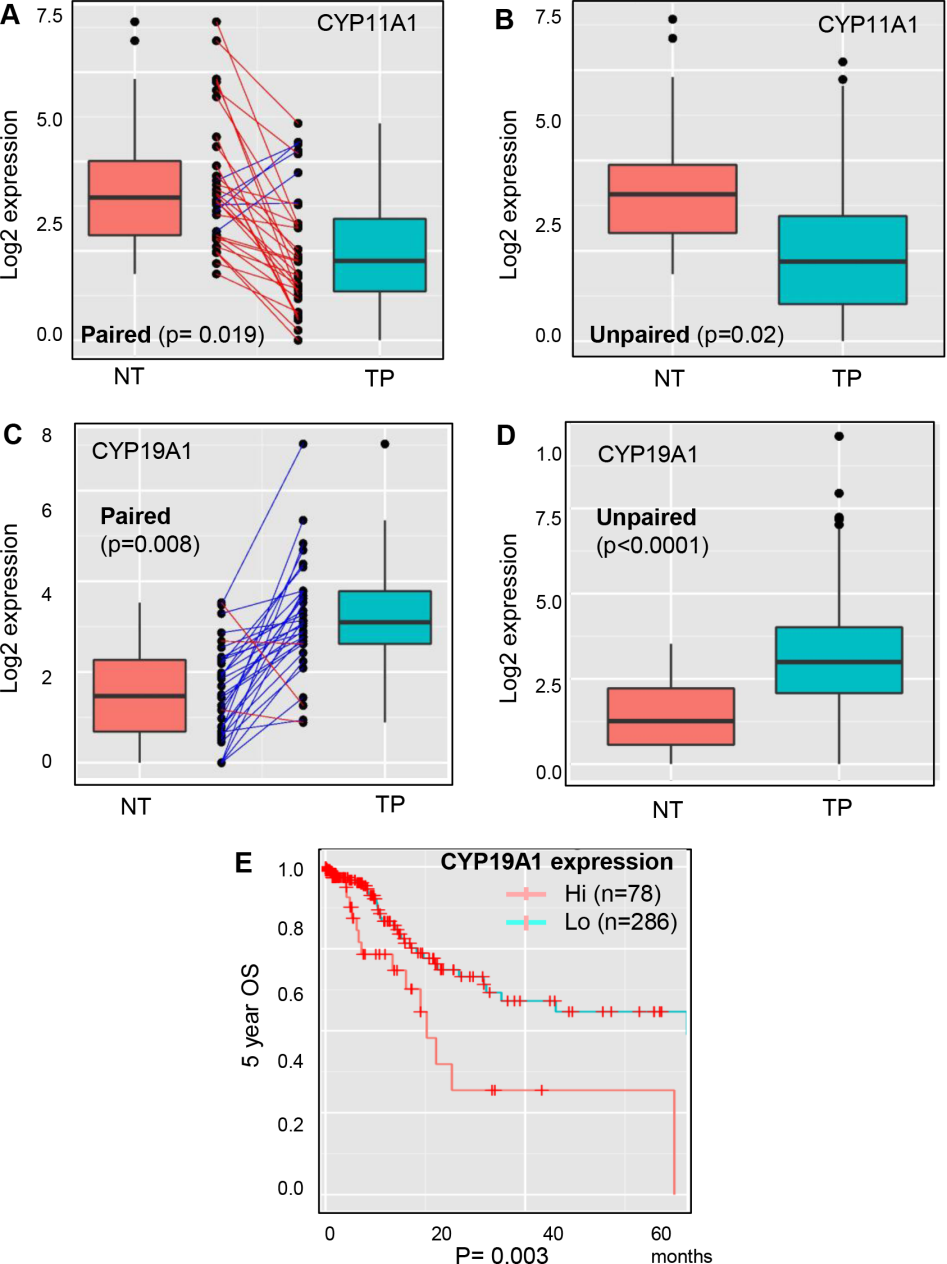
Expression analysis of CYP11A1 and CYP19A1 in non-tumor versus tumor parental lesions of GCa with DriverDB. v2 platform (**A**) Paired comparison of CYP11A1 expression in non-tumor (NT) and tumor-parental (TP) of gastric cancer patients. TP is significantly lower than NT, with a *p*-value 0.019. (**B**) Unpaired comparison of CYP11A1 expressions in NT and TP of gastric cancer patients. TP is significantly lower than NT, with a *p*-value 0.02. (**C**) Paired comparison of CYP19A1 expressions in NT and TP of gastric cancer patients. TP is significantly higher than NT, with a *p*-value 0.008. (**D**) Unpaired comparison of CYP19A1 expressions in NT and TP of gastric cancer patients. TP is significantly higher than NT, with a *p*-value less than 0.0001. (**E**) Validation of the database cohort association of CYP19A1 expressions to 5-year overall survival (OS) in GCa patients from the TCGA database. The patients were divided to two groups: hi (high expression; *n* = 78), and lo (low expression; *n* = 286). The logrank test *p*-value = 0.003.

The analysis of steroidogenic lipidomes revealed that CYP11A1 and CYP19A1 are progression dominant genes in various categories of GCa patients. Furthermore, we implemented TCGA data to estimate their expressions in non-tumor (NT) versus tumor parental (TP) in GCa patients. It was seen that CYP11A1 is lower (Figure 6A; *p* = 0.019) but CYP19A1 is higher (Figure 6C; *p* = 0.008) in TP compared to their NT counterpart. In addition, the non-matched comparison also consistently found lower CYP11A1 (Figure 6B; *p* = 0.02) but higher CYP19A1 (Figure 6D; *p* < 0.0001) expressions in TP compared to the NT lesions. These data suggest that targeting CYP19A1 might have a better response in tumors compared to non-tumor gastric tissue. Finally, we weighted CYP19A1 expression to associate with another patient cohort from TCGA. The data clearly demonstrated that high CYP19A1 expression is linked to poor overall survival compared to in low expression (Figure 6E).

### Targeting CYP19A1 as novel gastric cancer therapy

**Figure 7:**
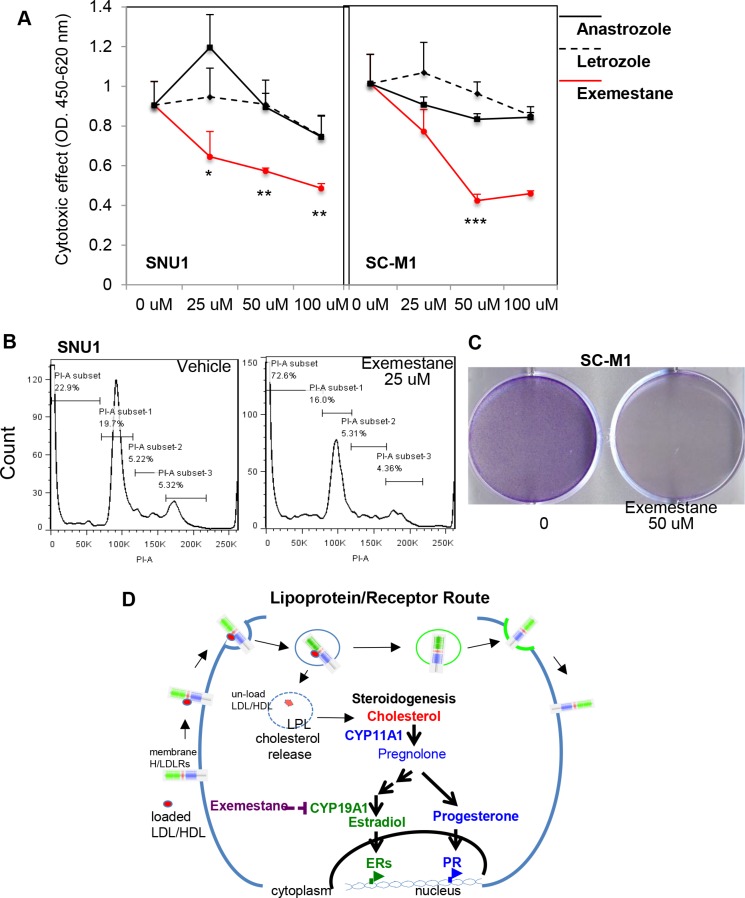
Targeting CYP19A1 with exemestane is novel therapy for GCa (**A**) Cytotoxic analysis of CYP19A1 inhibitors (Anastrazole, Letrozole, and Exemestane) in SNU1 (left panel) and SC-M1 (right panel) cells. The cytotoxic effect can be observed with exmestane, but not anastrazole and letrozole treatments. (**B**) Apoptotic cells was dramatically increased in SNU1 cells in 25 μM exemestane (right panel) compared with vehicle (left panel) treatments. (**C**) Long-term exemestane (25 μM; 7-days) can totally suppresses colony formation in SC-M1 cells compared to vehicle treatments. (**D**) Schematic illustration of L/R route to steroidogenesis in GCa cells. The cell membrane L/R route complex (LDLR, LRP6, SR-B1 and LPL) shuttles extracellular cholesterol carriers (e.g., LDL or HDL) into cancer cells. LPL is the catalysis to release cholesterol into cells, and receptors are then recycled back to the cell membrane. Increased cellular cholesterol provides resources for steroidogenesis, particularly CYP11A to increase progesterone or CYP19A1 to increase estradiol for PR or ESRs, respectively. Activated receptors could then alter genome-wide transcriptomes to promote cancer progression. Targeting steroidogenesis, e.g., CYP19A1 by Aromatase Inhibitors, exemestane, might be an effective therapeutic regimen in GCa patients.

In order to test that targeting CYP19A1 could be an effective therapy for GCa, three CYP19A1 inhibitor were applied on SNU1 and SC-M1 human GCa cell lines. As showed in Figure 7A, the type I CYP19A1 inhibitors (non-steroidal; Anastrazole and Letrozole) could not produce obviously cytotoxic effect within 48-hr culture. However, the type II CYP19A1 inhibitor (irreversible; Exemestane) exhibited drastically cytotoxic effect within 100 mM treatments (Figure 7A). Since SNU1 and SC-M1 are two distinct cell types (SNU1 is non-attached, while SC-M1 is attached to culture dish), we examined long-term Exemestane effect using sub-lethal dose (7-days; 25 mM) with flow-cytometry (Figure 7B; on SNU1) or colony formation (Figure 7C; on SC-M1) assays. We observed sub-lethal dose Exemestane treatment could significantly increase apoptosis of SNU1 cells (sub-G0 population from 23% to 73%; Figure 7B), while totally suppress SC-M1 colony-formation (Figure 7C).

Together, the data in Figures 5 and 6 strongly suggest that CYP19A1 could be the progression confounder in GCa patients. Targeting CYP19A1 with Exemestrane could be effective therapeutic agent for GCa.

## DISCUSSION

The pathophysiological impact of sex steroidogenesis in gastric cancer has been reported for decades; however, progress has been slow due to controversies. By taking advantages of recently developed web database and data mining strategy, we were able to approach to this question on the level of a whole pathway, rather than being limited to a few genes. We found that LDL and HDL import through their receptors via an outside-in cholesterol-shuffling route, and then cholesterol is unloaded by LPL (L/R route) into gastric tumors. The consequences of cholesterol uptake from L/R route favor the catalytic cascade of steroidogenesis enzymes to generate ligands for ESR1, ESR2 and PR. The algorithmic analysis and cancer driver analysis on steroidogenic lipidomes found that CYP19A1 is considered a landmark enzyme affecting disease prognosis, and is considered a valuable target for therapy. The finding has been summarized and illustrated in the Figure 6F. There are several major impacts of this report is discussed as follow.

### Route specific tumor macroenvironmental regulation affecting tumor progression

Cancer macroenvironment is rarely discussed due to uncertain resources of the effecter. Solid tumors are suspected to behave as systemic metabolic dictators and control whole body homeostasis in an endocrine organ-like manner. Solid tumors and peripheral organs might interact in a regulatory-feedback and continually evolving manner during tumor development [26], and our bioinformatics analysis further supported this idea. We also demonstrated the possibility of “host-to-tumor” regulation by the L/R route and steroidogenic lipidome in GCa patients. The advantage of using this method included: 1. Directly analyzing the effect of gene expression on disease progression in large patient cohorts. 2. Clustering genes based on concept/pathway to be weighted in cancer progression using a proper algorithm. 3. Testing hypothesis-driven gene clusters with a web-based cDNA microarray meta-analysis in an unbiased and genome-wide nature, which is more relevant to disease progression.

Utilizing the advantages of this method, we revealed the importance of the L/R route in promoting GCa progression, and there are several supporting evidences. For example, one recent study by Guillaumond et al. demonstrated the effect of cholesterol chemotherapy sensitivity in pancreatic cancer [27]. Although the mechanism of LDL/LDLR route in pancreatic cancer chemosensitivity was not described, it provided direct evidence of L/R route in pancreatic cancer *in vitro*. Other work by Tamura et al. also claimed that with higher serum HDL-cholesterol levels, there was lower survival in surgery GCa patients [28].

It has been debated that lipid-lowering drugs, e.g., statin, could reduce cancer risks. However, meta-analysis results are against the effect of cancer prevention or survival benefits of statin in several cancers [29], including GCa [30]. It is suspicious that statin lowered freely circulating cholesterol, but the lipid context of lipoprotein is uncertain; therefore conflicting conclusions exist. Another possibility is that cholesterol is imported as a ligand to activate ERRα, thereby governing statin effects in cells [31], and this could be related to L/R route in cancer.

### Targeting CYP19A1 to fill unmet medical needs for GCa therapy

Due to high incidence and poor prognosis, primary tumor removal at early stages of GCa is the only possible curative treatment. However, most patients have unresectable or metastatic disease at diagnosis. In the early 1980s, fluorouracil chemotherapy was evaluated as an active agent for GCa therapy either alone or combined treatment after surgery [32]. However, low response rate (19%–48%) and tolerable toxicity (> 50% patients with other gastrointestinal malignancies) make fluorouracil chemotherapy usually serve as reference arm in randomized phase III trials (37). HER2 expression in GCa has received attention as a potential target for therapy with trastuzumab [33], and is standard in the treatment of HER2+ advanced GCa [34]. Unfortunately, there is not better adjuvant therapy for HER2– GCa patients. Although trastuzumab can be applied, relapse in patients is frequent, even when combined with chemotherapies [35, 36].

The value of targeting steroidogenic enzymes has been speculated. Cho et al. [37] surveyed the single nucleotide polymorphisms (SNPs) of steroidogenic enzymes for an association with GCa risks. They found that CYP19A1 SNPs affect GCa susceptibility, and suggested CYP19A1 mutations might be GCa driver genes. The work presented here directly linked CYP19A1 to patient survival, further proving the value of targeting. We found CYP19A1 might be a valuable targeting site in particular patient populations, e.g., surgery or HER+/− patients. Our study clear demonstrated that using human tolerable dose Exemestane (25 μM) in GCa cells results in efficient cytotoxicity. Goss et al. (2011) [38] reported that long-term use of Exemestane exhibited an excellent breast cancer prevention effect with limited systemic complication. Our study pointed that clinical use of Exemestane in GCa patients might have chance to be success in clinical settings.

## MATERIALS AND METHODS

### Kaplan meier plotter for gastric cancer patient OS analyzer

We analyzed GCa 5-year overall survival (OS) rates using web-base gene survival analyzer Kaplan Meier Plotter (http://kmplot.com/analysis/index.php?p=service&cancer=gastric) [21]. 5-year OS was assessed in all GCa cohorts stratified by median classifiers expression. Gastric cancer subtypes included all patients (non-classified; *n* = 876), surgery (*n* = 380), surgery and 5′FU treatment (5FU and surgery; *n* = 153), HER2– (*n* = 532) and HER2+ (*n* = 344). Input genes and classifiers are: AR (201272_at), PR (208305_at), ESR1 (205225_at), ESR2 (211120_x_at), LDLR (202068_at), LRP6 (205606_at), SR-B1 (201819_at), CYP11A1 (204309_at), HSD3B1 (204515_at), CYP17A1 (205502_at), HSD17 (205829_at), CYP19A1 (203475at) and SRD5A1 (204675_at).

### Scoring method of Hazard Rations (HR) summation to evaluate targeting value (HR score)

To calculate the gene cluster impact on different GCa conditions with KM plotter survival analyzer, we developed a formula (Formula 1):

$$\text{HR score}=(\text{Avg}.\text{of HR of gene sets})\text{=}\frac{\Sigma (\text{HRn}-1)\times (-\log_{10}(p-\text{value}))}{n}\times 100$$

The HR of each gene is minus one, to adjust the effect of genes, multiplied with negative log10 (*p*-value) to balance the importance of genes. The summed score is divided by the number of gene, and multiplied by 100 to get the HR score, or average HR of each gene. The threshold was 100 to indicate significance of gene clusters. HR scores > 100 can be indicated as significant to be targeted, whereas HR scores ≤ 100 indicate a less value to be targeted for GCa therapy.

To validate the working capacity of the algorithm, we applied it to glycolysis enzymes (known GCa promoting metabolism pathway) [39] in GCa patients using KM plotter. The gene identifiers used were: 202022_at (ALDOC), 210050_at (TPI1), 212581_x_at (GAPDH), 205736_at (PGAM2), 201231_s_at (ENO1L1), 201313_at (ENO2), 204483_at (ENO3), 222078_at (PKLR), 201251_at (PK3), 206952_at (G6PC), 201578_at (PODXL), 200697_at (HK1), 205936_s_at (HK3), 210976_s_at (PFKM), 201102_s_at (PFKL) and 202382_s_at (GNPDA). The resulting HR score of glycolysis enzymes was 576, which is consistent with published results that glycolysis promotes GCa disease progression.

### Gastric cancer patients from TCGA

Previously, we developed DriverDB (http://ngs.ym.edu.tw/driverdb), a database that incorporates > 9500 cancer-related RNA-seq datasets and > 7000 exome-seq. datasets from TCGA, International Cancer Genome Consortium (ICGC) and published papers [40, 41]. In DriverDB, there are 420 primary tumors and 37 adjacent normal tissues (including 34 normal-tumor pairs) in the gastric cancer dataset of TCGA. We validated the expression of indicated genes in non-tumor (NT) versus tumor parental (TP) in a paired or non-paired fashion. We used Student's *t*-test to compare the mean expression levels of genes between primary tumors and adjacent normal tissues, and used paired *t*-test for matched normal and tumor pairs samples. A *p*-value less than 0.05 was considered statistically significant.

### Cytotoxic effect with WST-1 assay

The WST-1 assay (Roche, US) was adapted from previous work [42]. Briefly, 10^3^ cells/100 μl/well were seeded in 96-well plates with DMEM in 10% FBS and were incubated for designated time periods (1, 2, 4, 6, 8 days). Then 10 μl of WST-1 solution was added to each well and cells were allowed to incubate at 37°C in an incubator for an hour. Cell viability was quantified by colormetric detection in an ELISA plate reader (BECKMAN COULTER PARADIGM^™^ Detection Platform) at an absorbance of 450 nm and 690 nm to generate an OD proportional to the relative abundance of live cells in the given wells.

### Apoptosis assay

The apoptosis assay was conducted as previously reported [43]. Briefly, 10^6^ cells were cultured in a 100-mm dish with or without 10 nM DHT for 24 hrs, trypsinized, washed, and then stained with fresh 5 μM propidium iodine (PI) in PBS. The dead cells were then detected by flowcytometry (BD, SLRII) and analyzed by FlowJo 7.6 software.

### Colony-forming assay

Colony-forming assays were performed as previously study reported [44]. Briefly, 1 × 10^4^ cells/dish were seeded onto 3.5-cm plates with DMEM in 10% FBS with various treatments for 7 days. After treatments, 1/3 of total volume of 10% formaldehyde solution was added to fix cells, which were then allowed to stain with Crystal Violet for 5 mins. After wash with PBA, the colonies were photographed.

### Availability of data and material

The datasets generated in the current study are available in the (http://kmplot.com/analysis/index.php?p=service&cancer=gastric) repository. All datasets in the current study available from the corresponding author on reasonable request.

## CONCLUSIONS

In this report, a web-based gene analyzer platform followed by a gene summation algorithm evaluated the importance of the biochemical process of cholesterol transporting, steroidogenesis, and sex hormones receptor action in GCa cancer survival. The result showed CYP19A1 is a potential targeting site for GCa therapy. A clinical trial using Exemestane in GCa patient is needed.

## References

[1] Thrumurthy SG, Chaudry MA, Chau I, Allum W (2015). Does surgery have a role in managing incurable gastric cancer?. Nat Rev Clin Oncol.

[2] Tan P, Yeoh KG (2015). Genetics and Molecular Pathogenesis of Gastric Adenocarcinoma. Gastroenterology.

[3] Bang YJ, Van Cutsem E, Feyereislova A, Chung HC, Shen L, Sawaki A, Lordick F, Ohtsu A, Omuro Y, Satoh T, Aprile G, Kulikov E, Hill J (2010). Trastuzumab in combination with chemotherapy versus chemotherapy alone for treatment of HER2-positive advanced gastric or gastro-oesophageal junction cancer (ToGA): a phase 3, open-label, randomised controlled trial. Lancet.

[4] Allum WH, Blazeby JM, Griffin SM, Cunningham D, Jankowski JA, Wong R (2011). Guidelines for the management of oesophageal and gastric cancer. Gut.

[5] Foo M, Leong T (2014). Adjuvant therapy for gastric cancer: current and future directions. World J Gastroenterol.

[6] Sun J, Song Y, Wang Z, Chen X, Gao P, Xu Y, Zhou B, Xu H (2013). Clinical significance of palliative gastrectomy on the survival of patients with incurable advanced gastric cancer: a systematic review and meta-analysis. BMC Cancer.

[7] Korenaga D, Orita H, Okuyama T, Kinoshita J, Maekawa S, Ikeda T, Sugimachi K (1998). Sex hormone-receptor-negative tumors have a higher proliferative activity than sex hormone-receptor-positive tumors in human adenocarcinomas of the gastrointestinal tract. Surg Today.

[8] Duell EJ, Travier N, Lujan-Barroso L, Boutron-Ruault MC, Clavel-Chapelon F, Palli D, Krogh V, Mattiello A, Tumino R, Sacerdote C, Rodriguez L, Sanchez-Cantalejo E, Navarro C (2010). Menstrual and reproductive factors, exogenous hormone use, and gastric cancer risk in a cohort of women from the European Prospective Investigation Into Cancer and Nutrition. Am J Epidemiol.

[9] Cronin-Fenton DP, Murray LJ, Whiteman DC, Cardwell C, Webb PM, Jordan SJ, Corley DA, Sharp L, Lagergren J (2010). Reproductive and sex hormonal factors and oesophageal and gastric junction adenocarcinoma: a pooled analysis. Eur J Cancer.

[10] Chandanos E, Lagergren J (2008). Oestrogen and the enigmatic male predominance of gastric cancer. Eur J Cancer.

[11] Kim JH, Boo YJ, Park JM, Park SS, Kim SJ, Kim CS, Mok YJ (2008). Incidence and long-term outcome of young patients with gastric carcinoma according to sex: does hormonal status affect prognosis?. Arch Surg.

[12] Wu CW, Chi CW, Chang TJ, Lui WY, P'Eng F K (1990). Sex hormone receptors in gastric cancer. Cancer.

[13] Kuru B, Ozaslan C, Yalman K, Camlybel M (2002). Serum progesterone levels in patients with gastric and colorectal cancers. Acta Chir Belg.

[14] Inutsuka S, Kodama Y, Natsuda Y, Kumashiro R, Maekawa T (1986). Serum testosterone level of patients with gastric carcinoma before and after gastrectomy. Cancer.

[15] Sah BK, Chen MM, Peng YB, Feng XJ, Yan M, Liu BY, Fan QS, Zhu ZG (2009). Does testosterone prevent early postoperative complications after gastrointestinal surgery?. World J Gastroenterol.

[16] Myant NB (1971). The transport and turnover of the plasma cholesterol. Biochem Soc Symp.

[17] Caruso MG, Notarnicola M, Cavallini A, Di Leo A (2002). 3-Hydroxy-3-methylglutaryl coenzyme A reductase activity and low-density lipoprotein receptor expression in diffuse-type and intestinal-type human gastric cancer. J Gastroenterol.

[18] Enjoji M, Kohjima M, Ohtsu K, Matsunaga K, Murata Y, Nakamuta M, Imamura K, Tanabe H, Iwashita A, Nagahama T, Yao K (2015). Intracellular mechanisms underlying lipid accumulation (white opaque substance) in gastric epithelial neoplasms: A pilot study of expression profiles of lipid-metabolism-associated genes. J Gastroenterol Hepatol.

[19] Guo E, Chen L, Xie Q, Chen J, Tang Z, Wu Y (2007). Serum HDL-C as a potential biomarker for nodal stages in gastric cancer. Ann Surg Oncol.

[20] Li Q, Birkbak NJ, Gyorffy B, Szallasi Z, Eklund AC (2011). Jetset: selecting the optimal microarray probe set to represent a gene. BMC Bioinformatics.

[21] Gyorffy B, Surowiak P, Budczies J, Lanczky A (2013). Online survival analysis software to assess the prognostic value of biomarkers using transcriptomic data in non-small-cell lung cancer. PLoS One.

[22] Storey SM, McIntosh AL, Huang H, Landrock KK, Martin GG, Landrock D, Payne HR, Atshaves BP, Kier AB, Schroeder F (2012). Intracellular cholesterol-binding proteins enhance HDL-mediated cholesterol uptake in cultured primary mouse hepatocytes. Am J Physiol Gastrointest Liver Physiol.

[23] Iacovelli R, Pietrantonio F, Maggi C, de Braud F, Di Bartolomeo M (2016). Combination or single-agent chemotherapy as adjuvant treatment of gastric cancer: A systematic review and meta-analysis of published trials. Crit Rev Oncol Hematol.

[24] Liu H, Chen X, Sun J, Gao P, Song Y, Zhang N, Lu X, Xu H, Wang Z (2014). The efficacy and toxicity of paclitaxel plus S-1 compared with paclitaxel plus 5-FU for advanced gastric cancer: a PRISMA systematic review and meta-analysis of randomized controlled trials. Medicine.

[25] Galdy S, Cella CA, Spada F, Murgioni S, Frezza AM, Ravenda SP, Zampino MG, Fazio N (2015). Systemic therapy beyond first-line in advanced gastric cancer: An overview of the main randomized clinical trials. Crit Rev Oncol Hematol.

[26] Lee YM, Chang WC, Ma WL (2016). Hypothesis: solid tumours behave as systemic metabolic dictators. J Cell Mol Med.

[27] Guillaumond F, Bidaut G, Ouaissi M, Servais S, Gouirand V, Olivares O, Lac S, Borge L, Roques J, Gayet O, Pinault M, Guimaraes C, Nigri J (2015). Cholesterol uptake disruption, in association with chemotherapy, is a promising combined metabolic therapy for pancreatic adenocarcinoma. Proc Natl Acad Sci USA.

[28] Tamura T, Inagawa S, Hisakura K, Enomoto T, Ohkohchi N (2012). Evaluation of serum high-density lipoprotein cholesterol levels as a prognostic factor in gastric cancer patients. J Gastroenterol Hepatol.

[29] Dale KM, Coleman CI, Henyan NN, Kluger J, White CM (2006). Statins and cancer risk: a meta-analysis. JAMA.

[30] Shimoyama S (2011). Statins and gastric cancer risk. Hepatogastroenterology.

[31] Wei W, Schwaid AG, Wang X, Chen S, Chu Q, Saghatelian A, Wan Y (2016). Ligand Activation of ERRalpha by Cholesterol Mediates Statin and Bisphosphonate Effects. Cell Metab.

[32] Fujii M, Kochi M, Takayama T (2010). Recent advances in chemotherapy for advanced gastric cancer in Japan. Surg Today.

[33] Ieni A, Barresi V, Rigoli L, Caruso RA, Tuccari G (2015). HER2 Status in Premalignant, Early, and Advanced Neoplastic Lesions of the Stomach. Dis Markers.

[34] Wada R, Hirabayashi K, Ohike N, Morii E (2016). New guidelines for HER2 pathological diagnostics in gastric cancer. Pathol Int.

[35] Chrom P, Stec R, Szczylik C (2015). Second-line Treatment of Advanced Gastric Cancer: Current Options and Future Perspectives. Anticancer Res.

[36] Boekhout AH, Beijnen JH, Schellens JH (2011). Trastuzumab. Oncologist.

[37] Cho LY, Yang JJ, Ko KP, Ma SH, Shin A, Choi BY, Han DS, Song KS, Kim YS, Chang SH, Shin HR, Kang D, Yoo KY (2012). Genetic susceptibility factors on genes involved in the steroid hormone biosynthesis pathway and progesterone receptor for gastric cancer risk. PLoS One.

[38] Goss PE, Ingle JN, Ales-Martinez JE, Cheung AM, Chlebowski RT, Wactawski-Wende J, McTiernan A, Robbins J, Johnson KC, Martin LW, Winquist E, Sarto GE, Garber JE (2011). Exemestane for breast-cancer prevention in postmenopausal women. N Engl J Med.

[39] Yuan LW, Yamashita H, Seto Y (2016). Glucose metabolism in gastric cancer: The cutting-edge. World J Gastroenterol.

[40] Chung IF, Chen CY, Su SC, Li CY, Wu KJ, Wang HW, Cheng WC (2015). DriverDBv2: a database for human cancer driver gene research. Nucleic Acids Res.

[41] Cheng WC, Chung IF, Chen CY, Sun HJ, Fen JJ, Tang WC, Chang TY, Wong TT, Wang HW (2014). DriverDB: an exome sequencing database for cancer driver gene identification. Nucleic Acids Res.

[42] Chung WM, Chang WC, Chen L, Chang YY, Shyr CR, Hung YC, Ma WL (2013). MicroRNA-21 promotes the ovarian teratocarcinoma PA1 cell line by sustaining cancer stem/progenitor populations *in vitro*. Stem Cell Res Ther.

[43] Ma WL, Jeng LB, Lai HC, Liao PY, Chang C (2014). Androgen receptor enhances cell adhesion and decreases cell migration via modulating beta1-integrin-AKT signaling in hepatocellular carcinoma cells. Cancer Lett.

[44] Chen L, Chang WC, Hung YC, Chang YY, Bao BY, Huang HC, Chung WM, Shyr CR, Ma WL (2014). Androgen receptor increases CD133 expression and progenitor-like population that associate with cisplatin resistance in endometrial cancer cell line. Reprod Sci.

